# EngageHealth: a mobile device application designed to deliver stroke rehabilitation exercises using asynchronous video recordings

**DOI:** 10.3389/fstro.2024.1418298

**Published:** 2024-09-16

**Authors:** Andrew J. Song, Leonel Lugo, Julie Muccini, Michael Mlynash, Maarten G. Lansberg

**Affiliations:** ^1^Stanford Stroke Center, Stanford University School of Medicine, Stanford, CA, United States; ^2^Outpatient Neurologic Rehabilitation Program, Stanford Health Care, Stanford, CA, United States

**Keywords:** mobile device application, stroke rehabilitation, exercise, motor recovery, motivation, therapy

## Abstract

**Background:**

Stroke survivors who receive more rehabilitation therapy achieve better functional outcomes. The amount of rehabilitation that patients receive is, however, limited due to constraints of the healthcare system.

**Objective:**

To assess whether EngageHealth, a mobile device application designed to deliver stroke rehabilitation exercises using asynchronous video recordings, increases the amount of outpatient rehabilitation in stroke patients and improves their upper extremity function and quality of life.

**Design:**

Prospective single-arm study consisting of a 2-week pre-intervention phase without EngageHealth followed by a 4-week intervention period with EngageHealth.

**Setting:**

Ambulatory care.

**Participants:**

Twenty-four stroke patients with upper extremity impairment were recruited at the Stanford Stroke Center outpatient clinic.

**Interventions:**

Participants were instructed to use the EngageHealth application daily.

**Main outcome measures:**

Adherence, user experience, and change in the upper extremity Fugl-Meyer (UE-FM), Quality of Life in Neurological Disorders (Neuro-QoL), and Stroke Impact Scale (SIS).

**Results:**

Of 23 participants, five (22%) used the application for 17 days, six (26%) used the application for 9–16 days, and 12 (52%) used it < 9 days. Sixty-three percent of participants would recommend the application to other stroke survivors, with fifty percent indicating they would continue using the application, if available. During the pre-intervention phase, there were no changes in hand function. During the intervention period, participants improved by 4 points on the UE-FM (*P* < 0.01), and 15 points in the hand-function domain of SIS (*P* = 0.03). Videos of participants' exercises were successfully recorded, allowing the clinician to review videos of the participants' completed tasks asynchronously. In-depth interviews revealed that participants viewed the EngageHealth application favorably, and that their perceived usefulness of the exercises affected their motivation.

**Conclusions:**

Use of the EngageHealth application in the home environment may improve upper extremity function in subacute/chronic stroke patients. Additional support strategies should be implemented in future studies to improve adherence. These findings from a prospective single-arm study, support the design of a randomized controlled trial to determine the efficacy of long-term use of the EngageHealth application.

## 1 Introduction

Stroke is the leading cause of acquired adult disability (Langhorne et al., 2009). As many as 80% of patients experience weakness after stroke (Langhorne et al., 2009). Improvements in motor function can increase patients' ability to perform activities of daily living (ADLs). A recent study showed a positive association between time spent on therapy and clinical outcomes, with increased recovery among stroke survivors who receive more therapy (Lohse et al., 2014). However, therapeutic options for motor recovery are limited by various factors. Access to long-term daily intensive rehabilitation services is constrained by high costs and a lack of neuro-rehabilitation therapists (Strilciuc et al., 2021; Bernhardt et al., 2023). Logistical barriers, such as transport to rehabilitation appointments, can pose challenges for stroke survivors (Tran et al., 2024). Due to insurance limitations, patients may be ineligible to receive long-term therapy (Medford-Davis et al., 2016). Furthermore, depression and fatigue among stroke survivors can lead to decreased motivation to continue performing exercises long-term (Towfighi et al., 2017). Thus, there is a need to increase the amount of outpatient rehabilitation in stroke patients with weakness to improve motor recovery.

Technology has been used to increase the dose of stroke rehabilitation that patients can receive. Traditionally, physical and occupational therapists prescribe at-home exercises to stroke patients designed to improve their recovery. Recently, therapists have transitioned to using telehealth platforms to try to increase the amount of therapy (English et al., 2022). Additionally, robotic devices have been designed to increase recovery through administering high dose repetitive movement therapy under the supervision of a healthcare professional (Lansberg et al., 2022; Hong et al., 2024). However, these methods are limited by high costs, low compliance, and lack of accessibility (Clark et al., 2019; Mehrholz and Pohl, 2014). Several mobile device applications have been developed to target education, self-care, and rehabilitation among stroke survivors (Cao et al., 2024). Due to the widespread availability of mobile devices, such as smartphones and tablets, mobile device applications can be an effective way to increase the amount of rehabilitation after stroke (Szeto et al., 2023).

We developed the EngageHealth digital platform at Stanford to provide patients with a rehabilitation tool that (1) offers exercises specifically designed to aid in stroke recovery, (2) is portable so that exercises can be performed anywhere, and (3) records asynchronous videos of patients while they perform exercises, which can be later reviewed by clinicians. While other stroke mobile rehabilitation tools have been developed commercially, few of them meet these criteria (Cao et al., 2024; Szeto et al., 2023). Using a mobile device or small tablet, EngageHealth users can participate in several daily exercises assigned by a therapist. After viewing a brief video demonstration of the exercise, the participant performs the exercise and records a video of their performance, which is saved for asynchronous review by the therapist. The device provides notifications to nudge patients to complete their exercises and tracks their progress over time.

The purpose of the study was to assess participants' adherence and the efficacy of the EngageHealth application for home-based therapy among stroke patients with upper extremity impairment and a history of stroke. We hypothesized that the application is easily adopted by patients, increases their dose of rehabilitation, and promotes motor recovery.

## 2 Methods

### 2.1 Study design

We conducted a single-center, prospective, single-arm cohort study using the EngageHealth application for home-based upper extremity rehabilitation among patients with upper extremity impairment after stroke. We used a mixed-methods approach to collect quantitative data in the form of assessments and questionnaires, and qualitative data in the form of follow-up interviews. We obtained ethics approval from our institutional review board and informed consent from all participants prior to enrollment.

### 2.2 Study population

Consecutive patients who met eligibility criteria were prospectively enrolled at the Stanford Stroke Center. Inclusion criteria were (1) age 18 or older, (2) history of ischemic or hemorrhagic stroke within 1 year prior to enrollment, (3) upper extremity impairment with history of having received outpatient rehabilitation therapy, (4) provided informed consent, and (5) had access to a smartphone. Exclusion criteria were cognitive deficits, language difficulties (i.e., severe aphasia), or psychosocial difficulties that could impede participation. Patients included in analysis (per-protocol population) were those who provided consent and were given access to the application.

### 2.3 EngageHealth application

**Figure 1:**
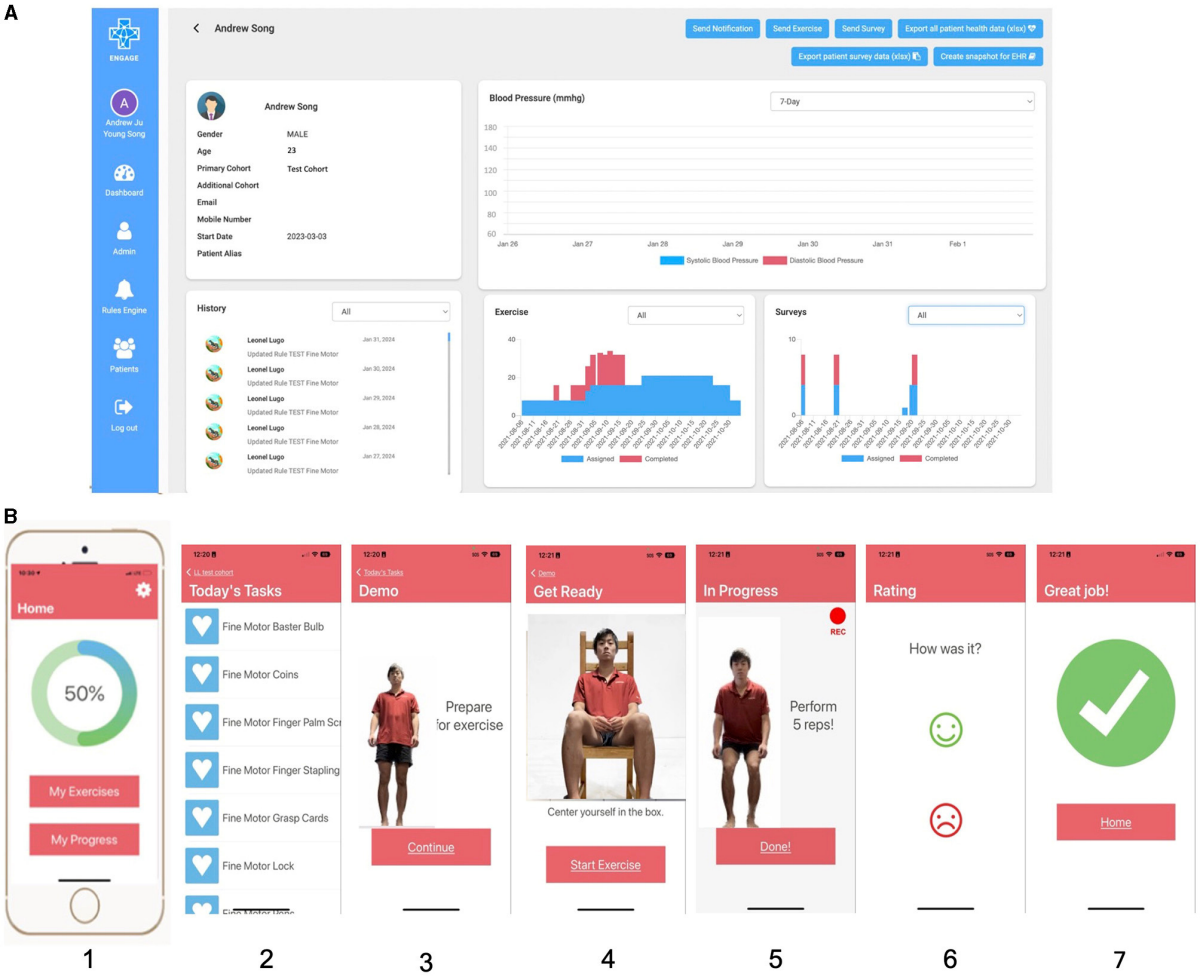
Screenshots of the EngageHealth application. **(A)** Shows the healthcare provider view of each patient on the computer. While there is a blood pressure tracking feature, blood pressures were not recorded for patients due to COVID-19 restrictions. **(B)** Shows the patient/user view of the application on their mobile device. In order from panel 1–7 is the home screen, list of assigned exercises/tasks, demonstration of the exercise/task, positioning of the user in the camera frame, video recording of user performing the exercise/task, rating of the exercise/task, and end screen.

EngageHealth is a Smartphone application designed at Stanford to help patients sustain motivation and empower them to increase their dose of home rehabilitation by connecting them with their therapist who can virtually assign rehabilitation exercises (Figure 1). On the EngageHealth provider platform, physical or occupational therapists can assign a set of daily exercises depending on their patient's level of weakness. After viewing a brief video demonstration of the exercise or activity of daily living task, patients perform the exercise/task while recording a video of themselves performing the activity. The video is automatically shared with their therapist who can review it asynchronously. Therapists can send notifications to their patients' mobile devices to nudge them to perform the daily exercises, or provide feedback based on their review of the video recordings. Patients can track their progress on the application.

For this study, exercises and activities of daily living tasks from a standard of care exercise program were prescribed to patients with upper extremity weakness. Exercises included stretching, active range of motion exercises, activities of daily living tasks, and fine motor activities with props for subjects with mild weakness. Fine motor and gross motor hand-based tasks included picking up pens and coins, rotating dials on a lock, and squeezing a spray bottle. Individuals with moderate to severe weakness, as determined by their medical charts, were assigned to a cohort receiving stretching exercises and activities of daily living tasks focusing on shoulder, elbow, and gross stabilization of the affected hand. A set of 14 exercises were released for the first 2 weeks of the intervention period, and a new set of 14 exercises were released for the second 2 weeks. Participants in each weakness group were prescribed the same set of 14 exercises daily.

### 2.4 Study timeline, procedures, and intervention

**Figure 2:**
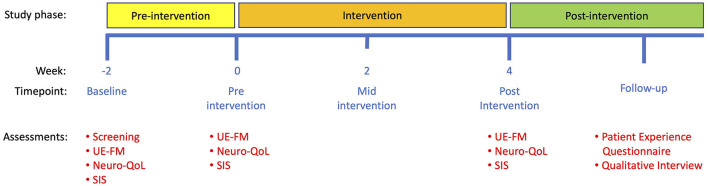
Study timeline. Note that a baseline visit was conducted 2 weeks before the start of the intervention to confirm that motor and functional stability was stable. Subsequent study visits took place at 0 weeks (pre-intervention) and 4 weeks (post-intervention). Patient Experience Questionnaires and qualitative interviews took place after the final post-intervention visit. UE-FM, upper extremity Fugl-Meyer assessment; Neuro-QoL, quality of life in neurological disorders assessment; SIS, stroke impact scale.

Participants underwent three visits over 6 weeks, consisting of a 2-week pre-intervention period and a 4-week intervention period (Figure 2). UE-FM assessments were conducted remotely on a synchronous secure Zoom call with two raters (JM, LL) scoring the assessment in real time. At the baseline visit 2 weeks before the intervention period (-2 weeks), after obtaining informed consent, an occupational therapist administered the UE-FM remotely, and participants completed the Neuro-QoL and SIS questionnaires. At the pre-intervention visit (week 0), participants repeated the UE-FM remotely to assess motor and functional stability and completed an additional set of Neuro-QoL and SIS questionnaires. After confirming motor and functional stability, all assessment measures at the baseline and pre-intervention visits were averaged and combined into a single “Pre-Intervention” score. Participants were then shown how to install and navigate the EngageHealth application on their personal devices. During the intervention period, the occupational therapist assigned participants daily exercises on the EngageHealth application. Participants were instructed to perform all assigned exercises daily through the intervention period and were contacted by a research coordinator weekly to assess adherence and to troubleshoot problems using the application if necessary. On average, notifications were delivered once per week to participants' devices to nudge them into performing their daily exercises. Participant data on the number of completed exercises and days of use were automatically recorded and stored on the EngageHealth platform. Some of participants' exercise videos were reviewed by the occupational therapist to assess the quality of their movements.

At the close-out visit (week 4), participants performed the final UE-FM remotely, completed the Neuro-QoL and SIS questionnaires, and were instructed on how to uninstall the application. All assessment measures at the close-out visit were labeled as the “Post-Intervention” score. We then followed up with each participant via telephone to administer a brief Patient Experience Questionnaire that included self-reported measures of (1) likelihood of recommending the application to other stroke survivors, (2) likelihood of continuing to use the application, (3) effectiveness of notifications, (4) design of exercise demonstrations, (5) degree to which exercises suited their needs, (6) clarity of instructions, (7) usability, (8) reliability, and (9) whether the application met their rehabilitation needs, each rated on a 5-point Likert scale (Supplementary Figure 1). In-depth semi-structured interviews were subsequently conducted with participants to gain a more detailed understanding of their recovery processes and experiences using the EngageHealth application. Interviews were ~1 h in duration.

### 2.5 Outcome measures

Our primary feasibility outcome was adherence, defined as number of days participants actively chose to use EngageHealth (Mir, 2023). Adherence data was automatically collected on the EngageHealth application. We categorized participants into three groups based on degree of adherence: (1) adherence of ≥17 days of use, (2) adherence of 9–16 days of use, and (3) adherence of ≤ 8 days of use. To count as 1 day of use, the participant must have completed all exercises assigned on that given day. For this categorization, we did not consider the quality of the exercises performed.

Our secondary outcome measures were changes on the Upper Extremity Fugl-Meyer (UE-FM) and on the Ability to Participate in Social Roles and Activities Domain of the Quality of Life in Neurological Disorders (Neuro-QoL) scales during the 4-week intervention phase (Kozlowski et al., 2016; Gladstone et al., 2002). Using conversion tables, raw scores from the 8-item Neuro-QoL short forms were manually translated into *T*-scores that are standardized with a mean of 50 and a standard deviation of 10, in reference to the U.S. general population (Cella et al., 2012). A higher score represents a better ability to participate in social roles and activities. Lastly, we assessed change in participants' self-reported measures of their disability and health-related quality of life after stroke on three domains of the Stroke Impact Scale (SIS), version 3.0: strength, activities of daily living and instrumental activities of daily living (ADL/IADL), and hand function (Mulder and Nijland, 2016). The strength domain includes four questions, ADL/IADL 10 questions, and hand function five questions, each rated on a 5-point Likert scale with a standardized maximum score of 100. Higher scores indicate a better quality of life.

### 2.6 Qualitative interview procedure

Qualitative research methods were in the form of in-depth semi-structured interviews (Emmerson et al., 2018). Narrative Inquiry methods were employed to craft questions that amplify the perspectives and lived experiences of stroke patients, and were cross-validated with three other researchers to craft neutral questions and rephrase any leading questions (Miles et al., 2020). Supplementary Table 1 shows an overview of the interview structure. We first asked general questions about their stroke journey and recovery process. We then asked follow-up questions about their questionnaire answers to investigate how their specific experiences may have influenced their perception of the application. Interviews with participants were conducted remotely over Zoom, and the audio and video were recorded. Interviews were then transcribed verbatim and all identifying information were removed.

Two rounds of coding were employed to all 11 interview transcripts using NVivo 14 qualitative data analysis software by AJS. The first round of coding included a set of descriptive, deductive codes to capture a basic understanding of a passage of qualitative data (Miles et al., 2020). These codes were formed based on predetermined notions of stroke recovery and patients' responses to the Patient Experience Questionnaires. The second round of coding included primarily inductive, interpretive codes. Inductive and *in-vivo* codes were prioritized during this second round of coding to preserve the authenticity of each patient's voice and to most accurately portray the patients' underlying thought processes. Following each round of coding, analytic memos were written. These codes were then iteratively categorized into more general subthemes and themes (Saldaña, 2016). Codes, subthemes, and themes were discussed and validated by the other researchers on the team.

### 2.7 Statistical analysis

Mann-Whitney *U*-tests were conducted for group comparisons between demographic groups in Table 1, and Kruskal-Wallis test between the three adherence groups in Table 2. Wilcoxon Signed-Rank tests were conducted for comparisons between timepoints in Table 3. Strength of correlation was assessed with Spearman's coefficient. All statistical tests were two-sided and deemed statistically significant at *P* < 0.05. Statistical analyses were conducted using IBM SPSS version 28 (IBM Corp, 2021).

**Table 1:** Baseline characteristics of study population.

	**Per-protocol population (*n* = 23)**	**Completed study (*n* = 16)**
**Demographics**		
Mean age, years (SD; range)	53.3 (15.1; 18–74)	49.9 (16.6; 18–74)
Male, *n* (%)	15 (65)	10 (62.5)
**Race**		
White, *n* (%)	11 (47.8)	8 (50)
Black, *n* (%)	1 (4.3)	1 (6.3)
Asian, *n* (%)	11 (47.8)	7 (43.8)
**Clinical characteristics**		
Mean time from stroke, months (SD; range)	34.5 (62.6; 3.0–269.9)	26.6 (66.3; 3.0–269.9)
No. with dominant affected hand (%)	10 (43.5)	7 (43.8)
Mean Baseline UE-FE score (SD; range)	34.1 (17.9; 4–57)	37.5 (16.9; 5–57)

The UE-FM score has a maximum score of 66 and a higher score indicates better function.

UE-FM, upper extremity Fugl-Meyer Assessment; Neuro-QoL, Quality of Life in Neurological Disorders Assessment; SIS-strength, strength domain of the stroke impact scale; SIS-ADL/AIDL, Activities of Daily Living (ADLs) and Instrumental Activities of Daily Living (IADLs) domain of the stroke impact scale; SIS-hand function, hand function domain of the stroke impact scale.

**Table 2:** EngageHealth adherence through 4-week intervention period.

	**Per-protocol population (*n* = 23)**	**Adherence ≥17 days (*n* = 5)**	**Adherence 9–16 days (*n* = 6)**	**Adherence ≤ 8 days (*n* = 12)**
Mean total days of use (SD)	8.7 (8.3)	22 (3.7)	10.7 (1.6)	2.2 (2)
Mean total number of exercises completed (SD)	70.6 (76.5)	191.8 (54.1)	81 (24)	14.8 (16.8)
Baseline FM score (SD)	34.1 (17.9)	37.5 (16.3)	35.8 (18.0)	31.8 (19.6)

One participant was not given intervention due to suffering an injury and was excluded from adherence analysis. Adherence ≤ 8 days group includes seven participants who were refused/declined or were lost to follow-up. All participants were prescribed the same number of exercises daily.

**Table 3:** Motor, functional, and quality of life assessments pre-intervention and change post-intervention.

	***n***	**Pre-intervention**	**Median change post-intervention**	***P*-value**
Median UE-FM Score (IQR)	16	40.8 (25.5–51.0)	4.0 (−0.3–6.0)	< 0.01
Median Neuro-QoL Score (IQR)	12	42.3 (39.9–46.3)	2.8 (−2.4–3.9)	0.38
Median SIS-strength (IQR)	12	46.9 (35.9–57.8)	0.0 (−10.2–4.7)	0.64
Median SIS-ADL/IADL (IQR)	12	73.1 (54.1–84.4)	0.0 (−15.3–2.8)	0.18
Median SIS-hand function (IQR)	9	25.0 (20.0–47.5)	15.0 (7.5–27.5)	0.03

Values are expressed as median (interquartile ranges). The UE-FM score has a maximum score of 66 and a higher score indicates better function. Neuro-QoL were converted to a T score which has a mean of 50 and SD of 10, in reference to the U.S. general population, and a higher score reflects a higher quality of life. Stroke Impact Scale has a standardized maximum score of 100, with higher scores indicating a lower difficulty performing a specific task and a better quality of life. Only participants with both pre-intervention and post-intervention measures were included in this analysis.

UE-FM, upper extremity Fugl-Meyer Assessment; Neuro-QoL, Quality of Life in Neurological Disorders Assessment; SIS-strength, strength domain of the stroke impact scale; SIS-ADL/AIDL, Activities of Daily Living (ADLs) and Instrumental Activities of Daily Living (IADLs) domain of the stroke impact scale; SIS-hand function, hand function domain of the stroke impact scale.

## 3 Results

### 3.1 Baseline characteristics

**Figure 3:**
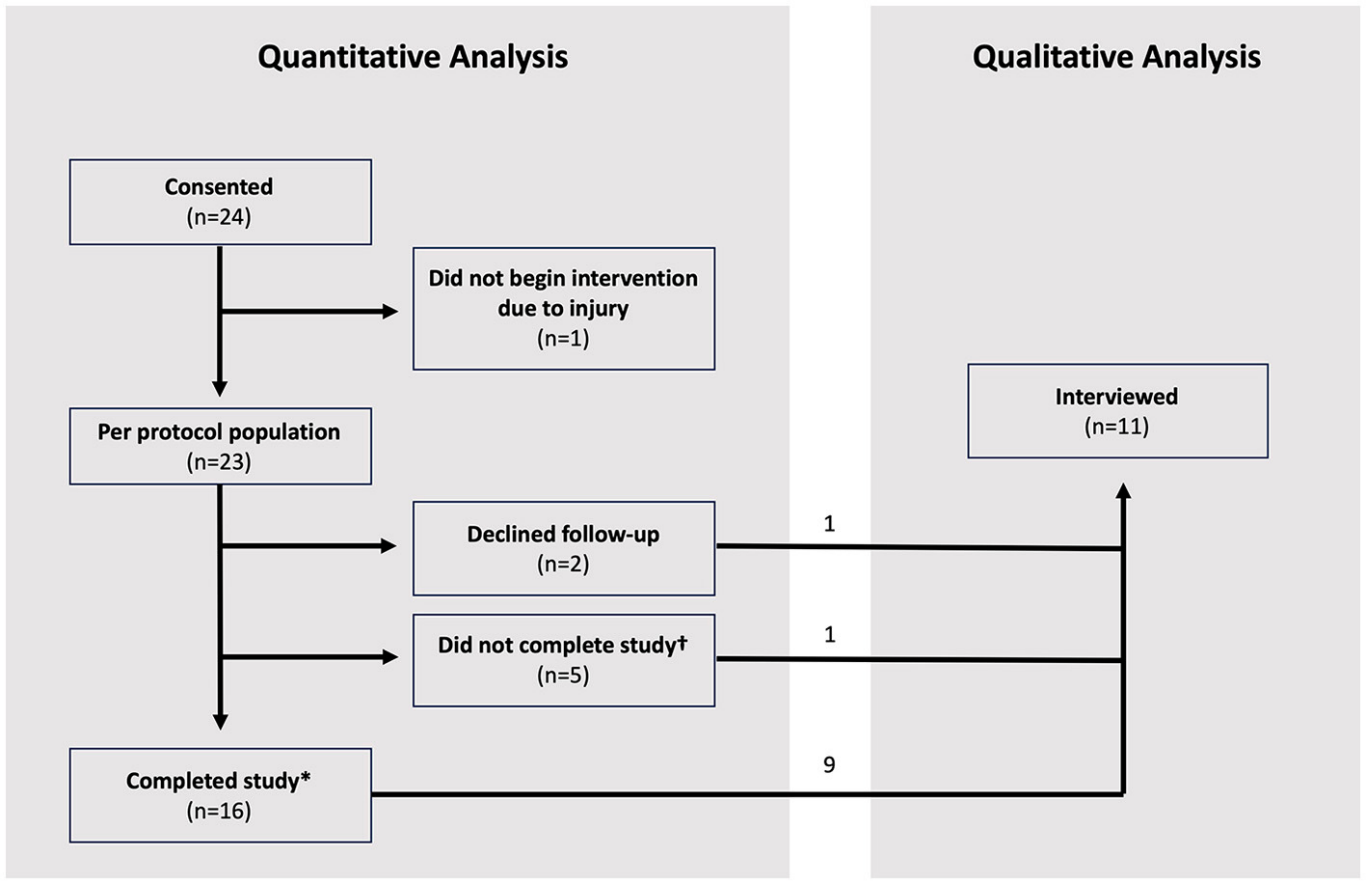
Study consort diagram of 24 participants. *Refers to participants who completed the final UE-FM assessment at week 4 post-intervention. ^†^Refers to participants who did not complete the final UE-FM assessment at week 4 post-intervention.

Twenty-four participants were enrolled between November 11, 2020 and May 5, 2022 during the COVID pandemic; baseline characteristics of the study population are shown in Table 1. One participant who was not given access to the application because he suffered an injury before the intervention period was excluded from the per-protocol analyses. Of the remaining 23 patients, two participants refused/declined to follow-up after getting access to the application, and five participants did not complete the final UE-FM assessment (Figure 3). The other 16 participants successfully completed the 4-week intervention period. The days of use and number of exercises completed were recorded for all participants. Video recordings of exercises were successfully recorded for eight of the participants.

### 3.2 Intervention adherence

Among the 23 per-protocol patients, adherence ranged from 0 days of use in four participants to as high as 28 days of use in one participant over the course of the 4-week intervention period. Five patients used the application ≥17 days (22%, *n* = 5/23); six used the application between 9 and 16 days (26%, *n* = 6/23), and 12 used the application ≤ 8 days (52%, *n* = 12/23). Of the seven participants who did not complete the final UE-FM assessment, one used the application between 9 and 16 days. The six other participants who did not complete the final UE-FM assessment used the application ≤ 8 days. The number of days each participant completed all assigned exercises and the total number of exercises completed over the course of the 4-week intervention period are summarized in Table 2 for the overall cohort and for the three subgroups.

Ten of 23 participants (43%) were older than 60 years of age (mean 67 ± 4 years vs. 43 ± 12 years for the remaining 13 participants). Adherence was compared between older (>60 years) and younger ( ≤ 60 years) participants to determine whether older participants were less inclined to use the application due to various possible factors (i.e., technological difficulties). There was no difference in the days of use between the older (median 6 days, IQR 11.3 days) and younger participants (6 days, IQR 10 days, *P* = 0.49), nor in the median total number of exercises completed between the older (42 exercises, IQR 8.8 exercises) and younger participants (43 exercises, IQR 3 exercises, *P* = 0.66). To test whether participants with higher baseline motor abilities may be more inclined to use the application, baseline UE-FM scores were compared between different adherence groups. There was no difference in the baseline UE-FM between participants with adherence of ≤ 8 days (median 34 points, IQR 34.8 points), participants with adherence between 9 and 16 days (median 36 points, IQR 22.8 points), and participants with adherence ≥17 days (median 48 points, IQR 23 points, *P* = 1.0).

### 3.3 Functional outcome

Functional outcomes are reported for the 16 participants who completed the 4-week intervention period. During the 2-week pre-intervention period, no changes were observed in their UE-FM scores (*P* = 0.82), Neuro-QoL (*P* = 1.0), and the strength, ADL/IADL, and hand function SIS measures (*P* = 0.55, *P* = 0.76, *P* = 0.49). Through the 4-week intervention period, the UE-FM scores improved by a median of 4 points (IQR 6.25 points, *P* < 0.01, Table 3). Improvement in UE-FM was weakly correlated with the number of days of use (*R* = 0.22, *P* = 0.42) and total exercises completed (*R* = 0.21, *P* = 0.43) on the application, pointing to a potential association with intensity of exercise, but this did not achieve significance in our limited cohort. The UE-FM scores for each participant at each visit are found in Supplementary Figure 2.

Twelve of the 16 participants who completed the study completed the Neuro-QoL assessments at their post-intervention visits. There was no overall change in the Neuro-QoL scores during the 4-week intervention period (*P* = 0.38).

Twelve of the 16 participants who completed the study completed the SIS questionnaires on strength and hand function, and nine completed the questionnaires on ADL/IADL at their post-intervention visits. During the 4-week intervention period, hand function improved by a median of 15 points (IQR 20 points, *P* < 0.05), but there was no change in the strength and ADL/IADL domains.

### 3.4 Participant experience

**Figure 4:**
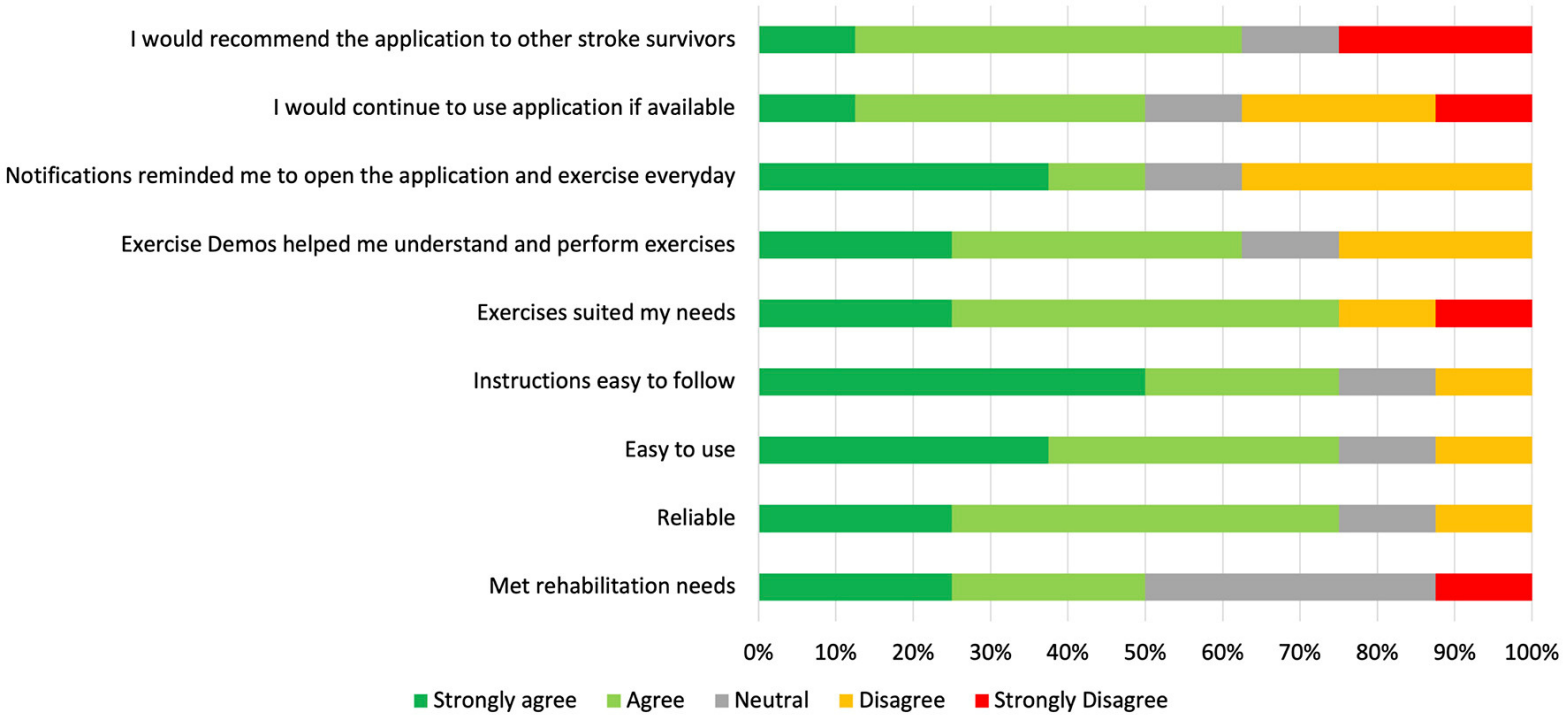
Results of Patient Experience Questionnaire Likert Scale Questions. Participants completed the survey during the follow-up period during the post-intervention phase of the study. Results of the surveys from eight participants who fully completed the study are reflected on this graph, with each participant representing 12.5% on the horizontal bars for each question. Of these eight participants, two users had an adherence level of 9–16 days, and six users had an adherence level of ≤ 8 days.

Results of the Patient Experience Questionnaires from eight of the participants are summarized in Figure 4. None of the users with adherence of ≥17 days (*n* = 0/5), 33% of users with adherence of 9–16 days (*n* = 2/6), and 50% of users with adherence of ≤ 8 days (*n* = 6/12) completed the Patient Experience Questionnaires. Most of the participants who completed the questionnaire would recommend the application to other stroke survivors (*n* = 5/8), believed that the exercises suited their needs (*n* = 6/8), and found the application to be easy-to-use and reliable (*n* = 6/8). Follow-up interviews of 11 participants were conducted to obtain a more in-depth and holistic understanding of participants' experiences using the EngageHealth application. Of these 11 participants, 36% had an adherence level of ≥17 days (*n* = 4/11), 36% had an adherence level of 9–16 days (*n* = 4/11), and 27% had an adherence level of ≤ 8 days (*n* = 3/11). Characteristics of interviewed participants are summarized in Table 4. Two major themes with associated subthemes emerged: (1) perceptions of EngageHealth Application and (2) characteristics of EngageHealth users (Supplementary Table 2).

**Table 4:** Participant interview characteristics.

**No**.	**Sex**	**Age**	**Race**	**Baseline UE-FM**	**Days of use**	**Study completion**
1	F	41	Asian	48	23	Completed
2	M	66	White	56	0	Declined follow-up
3	M	63	White	54	12	Completed
4	M	64	White	8.5	18	Incomplete
5	F	23	Asian	24	10	Completed
6	M	50	Black	36.5	13	Completed
7	F	74	White	49	19	Completed
8	M	73	Asian	26	20	Completed
9	M	43	White	32	3	Completed
10	F	32	White	50	28	Completed
11	M	47	Asian	55	2	Completed

UE-FM, upper extremity Fugl-Meyer Assessment.

#### 3.4.1 Perceptions of EngageHealth application

Based on the interviews, participants expressed a need for the EngageHealth application (Supplementary Table 3). Recent stroke survivors felt that the EngageHealth application served as a source of mental support while adjusting to their new lives after their stroke. Five users (45%) cited a lack of insurance coverage as a reason for discontinuing physical or occupational therapy after their stroke. Users felt that the EngageHealth application served as an alternative, convenient option for physical improvement. Seven users (63%) observed improvements in their physical abilities, four users (36%) noticed new abilities to perform daily tasks, and one user increased his confidence in existing abilities (9%). Movements and stretches while using the EngageHealth application provided relief for two users who suffered from other post-stroke conditions, including residual joint pain or migraines (18%). The EngageHealth application prompted users to move body parts that normally do not get regular exercise and revealed new areas of weakness. For example, one user discovered and re-activated “a part of the body which we are now trying to do more with in a very specific way” [Participant 9]. For participants who felt that they lacked time in their daily lives to dedicate solely to exercise, the EngageHealth application empowered them to manage their own stroke recoveries. One user stated, “When you come home, it's the app that guides you. It's actually good, because you don't have to go anywhere. It's just in the comfort of your bedroom…. It helped me with my hands because I can do however many reps I want” [Participant 1]. Four users actively adapted the exercises to suit their own abilities, target specific body parts, and integrate these movements into their daily activities (36%). Users said that being able to visualize their progress over time kept them motivated to use the application for longer periods (*n* = 4, 36%).

Users' suggested improvements to address challenges they encountered while using the application included:

1. Incorporate a broader range of exercises that are tailored to stroke survivors with a wider range of abilities in different stages of their recoveries.
2. Create practical exercises that can be easily translated and fit into stroke survivors' daily routines.
3. Provide users with clear instructions before each exercise on the specific objects required, the number of repetitions, and the optimal environment to perform the exercises. Prior to starting the exercises, the user could prepare a designated area with all the objects laid out beforehand, allowing the user to spend more time performing the exercises.
4. Design features that are accessible to stroke survivors who are hearing or visually impaired by implementing voice commands or using devices with larger screens, such as laptops or televisions.
5. Gamify the application to make exercise fun, where users can attain goals and enjoy the results. One user referenced DuoLingo, a language-learning application, which “rewards you for participating every day and it encourages you to maintain streaks and do it every day, even if only for 5 min” [Participant 5]. The constant reminders to complete daily challenges and participate in community leagues on DuoLingo successfully motivate users to challenge themselves and each other. There are frequent flashbacks to earlier lessons to prevent the user from losing abilities that they already acquired.

#### 3.4.2 Characteristics of EngageHealth users

**Figure 5:**
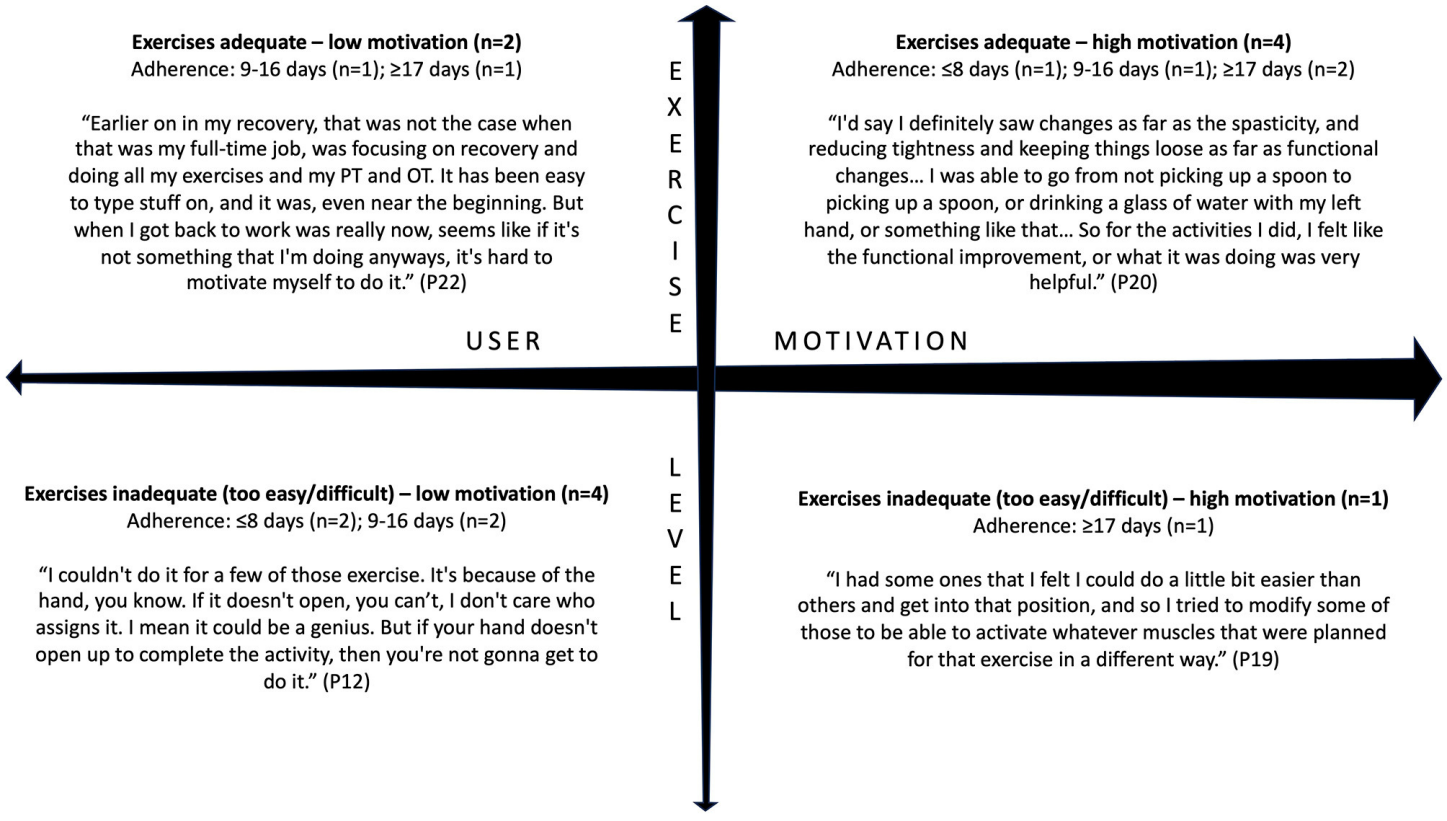
Interviewed users were classified into four categories based on their views of the EngageHealth application exercises and their motivation to use the application. Of the 11 interviewed users, four users had an adherence level of ≥17 days, four users had an adherence level of 9–16 days, and three users had an adherence level of ≤ 8 days.

While the complex nature of patient motivation is still not well-understood, qualitative methods using open-ended questions are an effective way to study motivation in the context of stroke rehabilitation (Maclean et al., 2000; Yoshida et al., 2021; Solbakken et al., 2023). Motivation was defined as the state that drives individuals to activate and sustain behavior toward a goal (Verrienti et al., 2023). In follow-up interviews, participants were asked about factors affecting their motivation to perform certain daily activities, including using the EngageHealth application. Thematic analysis revealed that participants' perceived usefulness of EngageHealth exercises affected their motivation to use the application (Figure 5). First, there was variability along an exercise level dimension. When asked how users perceived the exercise levels on the application, responses ranged from being adequate and suiting their needs to being inadequate and either too easy or too difficult. Second, there was variability along a users' motivation dimension, ranging from low to high motivation to exercise. Users were categorized according to the following quadrants: (1) users who felt that the exercises were useful but had low motivation due to external factors, (2) users who felt that the exercises were useful and were highly self-motivated, (3) users who felt that the exercises were either too easy or too difficult and had low motivation, and (4) users who felt that the exercises were inadequate yet were still highly self-motivated to exercise. Most users were classified into either the second quadrant, i.e., felt that the application exercises were useful and were highly motivated to exercise and improve (36%, *n* = 4/11), or the third quadrant, i.e., felt that the application exercises were inadequate and had low motivation (36%, *n* = 4/11).

In-depth interviews revealed the main reasons participants may have discontinued their use of the EngageHealth application. Users with high motivation held a more positive outlook toward their rehabilitation process, adapted exercises to suit their own abilities, and adhered to a strict schedule of using the application at a specific time each day (Supplementary Table 4). Motivated users set short-term, attainable goals and sought help from their caregivers to overcome technological or physical challenges. External and internal motivators are summarized in Supplementary Table 5. Users with low motivation stopped using the application because they felt that the exercises were not applicable to their individual needs (36%, *n* = 4/11). Despite feeling that the exercises were adequate, some users set unrealistic expectations for their recovery process and felt demotivated when met with the reality of their progress. Four users stopped using the application due to scheduling issues or lack of time after returning to work or school (36%). Overall, six of the users (54%) expressed a desire to continue using the application after the conclusion of the study.

## 4 Discussion

To assess the utility of the EngageHealth application in promoting stroke recovery, we used a mixed-methods approach that capitalizes on the unique strengths of quantitative and qualitative data. A quantitative research approach was used to assess the functional effect of the EngageHealth application and capture initial feedback in an unbiased manner. A qualitative research approach was used to accurately amplify the patient's voice regarding their perceptions of the EngageHealth application, without a priori hypothesis, and are particularly well-suited for addressing changes in medical device implementation (Miles et al., 2020).

This study shows that some stroke patients can use the EngageHealth application for home-based therapy without direct supervision by a therapist. After receiving brief instructions on how to navigate the application, participants used the application an average of 9 days and performed over 70 exercises overall, with some using the application for as many as 28 days during the 4-week intervention period. During the intervention period, participants improved their upper extremity function by a median of 4 points on the UE-FM and 15 points on the hand function domain of the SIS. These changes approach but do not meet the minimal clinically important differences of 4.25 points for UE-FM and 17.8 points for hand function of SIS (Lin et al., 2010; Page et al., 2012). While EngageHealth may improve specific motor functions, the impact of the application on overall quality of life is not known. Subsequent studies with larger sample sizes can investigate whether these changes persist over long-term periods of using the EngageHealth application. Changes in our study may not have met the minimal clinically important differences because more than fifty percent of participants had adherence of ≤ 8 days (*n* = 12/23).

Our study showed a trend suggesting that participants who used the application more frequently achieved greater improvements in UE-FM scores. Other studies have similarly found an association between increased adherence to exercises or device use and improved functional outcomes (Lohse et al., 2014; Scronce et al., 2023; Gunnes et al., 2019; Duncan et al., 2002; Burridge et al., 2017). There was also a trend that participants with ≤ 8 days of adherence had lower baseline UE-FM scores compared to participants with >8 days of adherence. This paralleled findings from the follow-up interviews which demonstrated that users who began the study with greater physical impairment encountered difficulties with performing exercises and were frustrated and unmotivated to continue using the application. Personalized sessions between users and their clinician to simplify movements or further tailor the set of exercises to their specific needs could increase users' motivation and improve overall experiences with the application. Additionally, support strategies are necessary to promote participants' adherence to interventions, especially among stroke patients who are not fully functional (Friedman et al., 2015; Bebbington, 1995). The lack of support strategies in our study for participants likely contributed to poor participant adherence. Future studies should implement support strategies to improve adherence, such as regular reminders and check-ins, progress feedback, and involvement of family members (Mahmood et al., 2022; Camarneiro, 2021).

Most participants who completed the Patient Experience Questionnaire indicated that they would recommend the EngageHealth application to other stroke survivors, felt that the exercises and demonstrations were useful, and found the application interface to be reliable and easy-to-use. However, all of the participants who completed the Patient Experience Questionnaire had adherence of ≤ 16 days and are likely not representative of the entire per-protocol population. Research suggests that exercising at least once per week can elevate mood (McDonnell et al., 2014). While participants who completed the Patient Experience Questionnaire did not fully adhere to instructions of daily exercise, completing the exercise routine even a few times per week may have been enough to provide an overall positive experience with the EngageHealth application. Follow-up interviews were conducted to gain a more holistic understanding of users' experiences and identify other factors that may have contributed to low user adherence. Interviewed participants discontinued the EngageHealth application due to the lack of exercises that suited their individual needs, unrealistic expectations for recovery, and scheduling issues due to the COVID pandemic, as opposed to the application design and features.

Follow-up interviews captured the application's positive impact on users' quality of life that were not observed in the Neuro-QoL or SIS scores. Seven participants observed improvements in recovery after using the EngageHealth application, and the benefits of the application extended beyond the functional or motor improvements observed in the UE-FM and SIS. These benefits included providing mental support, relieving pain, and empowering users to take control of their own recoveries. Improvement in the self-reported hand function domain of the SIS over the 4-week period reflected these sentiments.

Motivation in the context of stroke rehabilitation is defined as the underlying reason for patients' sustained efforts toward recovery and is affected by both intrinsic and extrinsic factors (Yoshida et al., 2021; Verrienti et al., 2023). Qualitative analysis of follow-up interviews showed that participants' perceptions of the EngageHealth application varied along an exercise level theme and a motivation theme. Most users viewed the exercises as adequate and were highly motivated or felt that the exercises were inadequate and were demotivated to use the application. These findings are in line with the Technology Acceptance Model, which postulates that users are more likely to adopt a new piece of technology based on two criteria: perceived usefulness and ease of use (Davis, 1989). This suggests that including a wider range of exercises tailored to users' specific needs could increase users' motivation to use the application.

Few other mobile device applications have been developed to deliver stroke rehabilitation to stroke survivors using exercise videos. One randomized controlled trial found that upper limb home exercise programs supported by video and automated reminders on electronic tablets were not superior to standard paper-based home exercise programs (Emmerson et al., 2017). On the other hand, another randomized controlled trial found that in-home pamphlets with a QR code for exercise videos improved exercise adherence, self-efficacy for exercise, and mobility gain but not basic activities of daily living (Chung et al., 2020). Improvements in UE-FM assessments observed in our EngageHealth study (median of 4 points) were less than other stroke mobile device applications (mean of 9.81 points), which include Rehabilitation Guardian, an application that provides continuing nursing care, and a home-based rehabilitation system that facilitates home exercises using a smartwatch (Cao et al., 2024; Xu et al., 2023; Chae et al., 2020). However, unlike EngageHealth, these interventions require synchronous monitoring of user data and additional expensive equipment.

There are potential drawbacks to the EngageHealth application. Stroke survivors must possess a smartphone or tablet to use the EngageHealth application, which can be expensive and inaccessible to the general population. However, with recent technological advances that lower the cost barrier for owning smart devices, the technological divide has decreased (Rotondi et al., 2020). Approximately 85% of adults in the United States own a smartphone, with rates similar across racial groups (Atske and Perrin, 2021). Another concern is that learning to use the application may be difficult for those who do not have prior experience with technology, such as older users. However, adherence did not vary across age groups. All participants who were interviewed had prior experience with smart device technology and found learning how to navigate the application easy. Finally, the application may be inaccessible to post-stroke survivors with visual or physical impairments. Follow-up interviews revealed that users with these impairments were able to overcome these challenges with caregiver assistance.

The EngageHealth application has many advantages. First, the mobile device application allows for at-home therapy, without the cost and logistics of travel to an in-person rehabilitation center. The EngageHealth application could also increase accessibility and consistency of therapy for patients in remote or rural locations far from therapy centers. Second, some of the participants successfully followed the application instructions to position the camera and record videos of themselves performing the exercises. These asynchronous video recordings could allow therapists to monitor their patients' progress, enhancing communication and improving patients' motivation to perform at-home exercises. Third, some short-term and long-term users could potentially benefit from the EngageHealth application. Six of the 11 interviewed users expressed their interest in continuing to use the EngageHealth application beyond the length of the study. For stroke survivors who regularly attend physical or occupational therapy sessions, therapists can integrate their exercises into the EngageHealth platform. For long-term rehabilitation, the EngageHealth application is a low-cost, alternative option for those who cannot afford in-person physical or occupational therapy because they either no longer qualify or lack insurance coverage. Fourth, the source code for the EngageHealth platform can be made publicly available online for developers to improve upon and implement the application in other medical contexts.

There are several limitations to our study. Since the study is not a double-blind randomized controlled trial, we cannot conclude that the changes observed were solely due to the EngageHealth application, as opposed to a placebo effect or traditional therapy. To address this, we confirmed no functional or motor changes in the 2-week pre-intervention phase prior to introducing the application to participants. Most of the participants chosen for this study no longer attended physical or occupational therapy, which could have confounded the results. Future studies with larger sample sizes can employ multivariate analyses to explore the effects of other potential confounding factors, such as prior experience with technology, severity of stroke, and baseline functional status. Second, although we observed a significant increase in UE-FM and SIS hand domain scores, changes in the other measures were not statistically significant. Since our study took place during the COVID-19 pandemic, restrictions presented additional difficulties in terms of remote UE-FM assessments and participant follow-up. Considering that only 16 of the 23 per-protocol participants fully completed the study and no power calculation was conducted to inform sample size, these results may be underpowered and would likely require larger sample sizes to be validated. Moreover, our strict inclusion criteria likely excluded patients who could benefit from using EngageHealth and limits the generalizability of our findings to the broader stroke patient population. Future studies could investigate whether using EngageHealth for longer periods beyond 4 weeks among a larger and more diverse participant group results in increased effect sizes, compared to other standard home exercise programs used by clinicians. Third, the definition of adherence categories was determined *post-hoc*, and a significant proportion of participants had adherence of ≤ 8 days (52%, 12 of 23 participants). Given that most of the interviews were conducted among participants with adherence of ≥17 days, our qualitative findings may not be representative of the larger cohort. Future studies should use our findings to predefine more precise adherence categories that account for stroke patients' need for supports and to investigate ways to improve user adherence to EngageHealth. Fourth, not all of the participants' videos were captured due to a glitch in the application system, and we were unable to evaluate the quality of all the participants' exercises. Of the participants' videos that were available, some participants demonstrated that they were capable of filming themselves performing exercises and functional tasks after review by an occupational therapist (JM; 62%, eight of 13 participants). Furthermore, follow-up interviews captured details about how participants' felt about following instructions to record videos and about the specific exercises assigned to them in terms of suitability and difficulty. Future studies that carefully review video recordings are required to determine whether support strategies can improve stroke patients' ability to film themselves performing tasks and complete the exercises. Fifth, we did not collect data on the provider side of the EngageHealth platform. Since our study focused on the patient side of the application, future studies could investigate how providers feel about using the EngageHealth application.

## 5 Conclusion

The EngageHealth platform, a mobile device application designed to deliver stroke rehabilitation exercises using asynchronous video recordings, can increase motivation and dose of rehabilitation in patients with upper extremity weakness and a history of stroke. Participants used the application twice per week, on average, and observed an improvement in functional recovery of their upper extremity and hand function over the course of 1 month. However, our study lacks necessary support strategies to improve adherence among users. These findings from a prospective single-arm study, support the design of future studies with long-term follow-up, larger sample sizes, and more diverse populations to improve user adherence and evaluate the efficacy of the EngageHealth application.

## Data Availability

The raw data supporting the conclusions of this article will be made available by the authors, without undue reservation.
