# Supplementary material for: EngageHealth: a mobile device application designed to deliver stroke rehabilitation exercises using asynchronous video recordings
Source: Front Stroke. 2024 Sep 16;3:1418298. doi: 10.3389/fstro.2024.1418298 (PMC12802795; doi:10.3389/fstro.2024.1418298)
Supplement: Supplementary file 1 [file Table_1.DOCX]

Supplementary Material

EngageHealth: a mobile device application designed to deliver stroke rehabilitation exercises using asynchronous video recordings

Andrew J. Song^1^, Leonel Lugo^1^, Julie Muccini^1,2^, Michael Mlynash^1^, Maarten G. Lansberg^1*^

*** Correspondence:** Maarten G. Lansberg: lansberg@stanford.edu

# Supplementary Tables and Figures

| **Supplementary Table 1.** Interview Guide | |
| --- | --- |
| **Themes** | **Questions** |
| Background | Can you describe your stroke journey and involvement in this clinical study? |
|  | Can you describe your recovery process since having your stroke? |
|  | Can you walk me through a typical day? |
|  | Do you have access to a caregiver? |
|  | Can you describe your experience with physical/occupational therapy? |
|  | Are there any activities in your daily life that you feel motivated to do? |
| Experience with EngageHealth Application | Can you describe your prior experience with technology? |
|  | Can you describe your experience with mobile device applications? |
|  | Can you walk me through a typical day of using the EngageHealth Application? |
|  | How do the EngageHealth exercises compare to rehabilitation clinic exercises? |
| Perceptions of EngageHealth Application | Have you experienced changes in your life after using the EngageHealth Application? |
|  | What advice would you give to new users of the EngageHealth Application? |
|  | If you were to use the EngageHealth Application again, would you do anything differently? |
|  | Looking into the future, what are your hopes and expectations for future recovery? |
|  | |

## Supplementary Tables

| **Supplementary Table 2.** Perceptions of EngageHealth Application and Characteristics of Users | |
| --- | --- |
| **Theme** | **Subtheme** |
|  | Unmet Need for a Mobile Device Application |
| Perceptions of EngageHealth Application | Benefits of EngageHealth |
|  | Suggestions for Improvement |
|  | Reasons for Discontinuing EngageHealth Use |
| Characteristics of EngageHealth Users | Mindsets of Compliant Users |
|  | Sources of Motivation |
|  | |

| Supplementary Table 3. Unmet need for a mobile device application and how EngageHealth satisfies the unmet need, with example quotes from users. | | | |
| --- | --- | --- | --- |
| Unmet Needs and Benefits of EngageHealth Application | | | |
| Unmet Need | Example | Benefit Addressing Unmet Need | Example |
| Lack of support | “For a person with a hemorrhage stroke, probably, for your research is just that, you are kind of alone. No, you have help, you have your parents, or you know, other loved ones, siblings, or what not. But no one understands your fight. So you kind of have to just like, you know just grit down and do it yourself because they cut off your therapy, so you don't have that support anymore. So you just on your own.” [Participant 12] | Source of mental support and guidance | “To know that you have some sort of support because, having their stroke is mentally challenging as well as physical. I would say that mentally, it kind of revs you up to try and accomplish the tasks that are on there to do.” [Participant 12]  “Having an app that showed me a video again, of exactly how it was supposed to be done… [was] quite helpful in reinforcing.” [Participant 5] |
| Lack of alternative recovery options | “In terms of chronic pain, there are very few options, and our systems are not well designed for it. Therefore having access to a program like [EngageHealth], where there is a structure for movement is very beneficial. It would be great if things like this were available to complement in-person training.” [Participant 9] | Improvements in physical abilities and pain relief | “I was able to go from not picking up a spoon to picking up a spoon, or drinking a glass of water with my left hand” [Participant 20]. |
| Expensive Therapy due to insurance policies | “[The hospital] stopped therapy because they said the insurance company won’t authorize more payments unless you're doing something new. They don't want to keep paying for the same activity for too long. They have to see some type of improvement and something new, something different in order to authorize payment. So once I heard that, I said, I need to figure out a way to be my own [trainer], because sooner or later these reimbursed sessions are going to go away. So I need to figure out myself what I need to do to continue to do my own rehab on my own.” [Participant 19] | Empower Users to manage own therapy | “I began to understand why those exercises were created. What was it in those exercises that would help me improve my recovery? That's how I adapted some of those movements.” [Participant 19] |
| Lack of time | “My life is boring. I just have to take care of the kids and then go to work. I don't, like I said, I don't have time to do stuff. I'm pretty much busy, so I don't even work out.” [Participant 1] | Convenient | “When you come home, it's the app that guides you. So it's actually good, because you don't have to go nowhere. It's just in the comfort of your bedroom.” [Participant 1] |

| **Supplementary Table 4.**  Mindsets of compliant users of EngageHealth, with example quotes from users. | |
| --- | --- |
| Mindsets of Compliant Users | |
| Mindset | Example |
| Acceptance of new life | “Now I’m in a stage of my life, where I have to accept what I am right now, and that I could never return to the normal me before. It's very hard to accept, because, you know, I’m being a nurse for 20 years now and that's the identity that I have. And now it was just taken away from me. So it hits me hard. It doesn't sink in, and it's slowly getting in, but I know I'll get there one day. I don't have a choice. Either I like it or not. This is my new norm now.” [Participant 1] |
| View of Recovery As a Journey | “It's been a journey. But I am recovering still. So that's good. I'm not plateauing.” [Participant 10] |
| Translation to Activities of Daily Living | “Try to integrate [exercises] into your ADL’s because that's what the app is, trying to make you do the things that is harder for you to do in terms of like exercising it. Like you know, the app, it's a starter. It teaches you how to do things by doing the exercises. So that's how I look at the app for my own perspective.” [Participant 1] |
| Short-Term Goalsetting and Realistic Expectations | “You start like 3 days straight, then 3 days become 5, and then one week, and then 2 weeks. You gradually get into it. You don't have to do all at once, right. Because most of that time the expectation is that you do it every day. But life happens. The reality of it all is, people cannot do what you tell them to do so the credibility is on the app, and then eventually it becomes, you know, a routine for you, then that's it. It will do you good.” [Participant 1] |
| Positive View of Exercise | “Whatever you can do on there, do more of it. The things you can’t do… don't focus on the one that you can’t do, focus on what you can do and do more of it. So if you're like for me, I was able to open a fridge and close it on the consistent level. So I do more of that than I do any of the other exercise, because I see progress with that. But I could do that and it was a struggle. But I do more of those than I do anything else. So whatever you can do a little bit better than other activities, do more of what you can do.” [Participant 12] |

| **Supplementary Table 5.**  External and internal motivators of EngageHealth users, with example quotes from users. | | | |
| --- | --- | --- | --- |
| External | | Internal | |
| Motivators | Examples | Motivators | Example |
| Family members | “Watching my parents and in-laws decline certainly is a motivator.” [Participant 5] | Hobbies | “I knew that trying to get these muscles activated regularly was what's important, so that kind of helped motivate me to try to get things working faster. I try to set a goals for myself in terms of at what point do I want to start playing golf again.” [Participant 19] |
| Video monitoring | “I mean since we're videoing, there's always that possibility someone was looking at it or would look at it. And that gave a little bit of sort of incentive.” [Participant 14] | Occupation | “I miss going back to work because of my disability. It's been 3 years now that I haven't gone to work.” [Participant 1] |
| Caregiver Support | “I think that before my stroke, problem solving was a different process than it is now for me, and I think just having another person there to talk it through and to troubleshoot with would have been, and was really helpful.” [Participant 22] | Progress | “It's a lot of work to maintain the function I have, but I also gain new function and new skills, and a lot of that, of course, is because of computers and the things they can do, but that's exciting. I'm more excited about life now than I've been at any time that I can recall.” [Participant 5] |
| Caregiver Encouragement | “Technologically she wouldn't have handled at the time, but also she didn't have the motivation. I had motivation because I wanted her to be able to cook. So yeah, it helps to have a caregiver who gets the benefit of her improvement. I don't think there's any way around that. For an older person who's had a stroke to get into a new application must be really hard.” [Caregiver of Participant 14] | Frustration | “For the physical side, I just want my hand to come back. It's really frustrating, not using my arm, and so I want to get it back.” [Participant 12] |

## Figures

**Supplementary Figure 1.**  Patient Experience Questionnaire given to the participants during the post-intervention period.

##
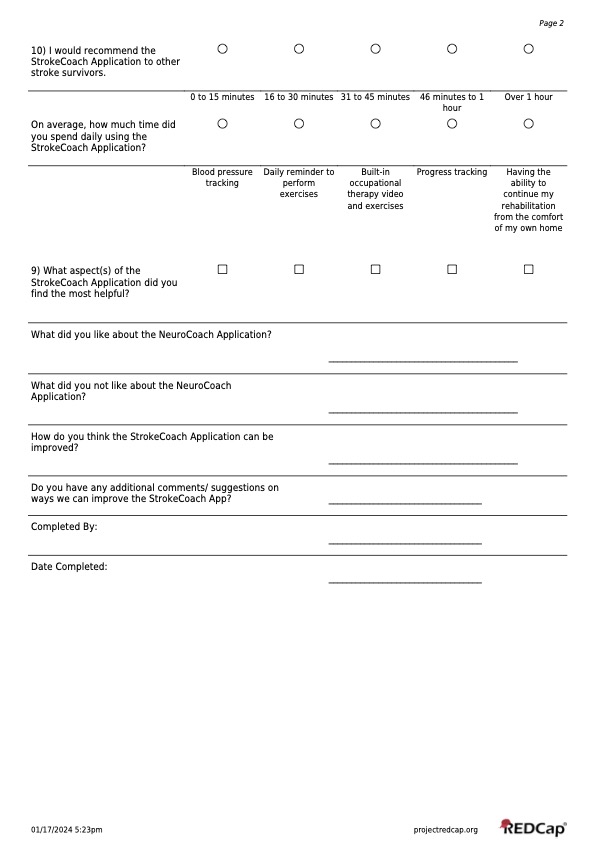


**Supplementary Figure 2.**  (A) Upper-Extremity Fugyl-Meyer (UE-FM) Assessment Scores for each per-protocol participant (n=23) at each time point of the study. Red denotes participants who did not complete the final UE-FM assessment at the post-intervention visit. Changes in UE-FM versus (B) days of application use and (C) number of exercises completed. Red lines denote regression lines and gray regions represent 95% confidence intervals.

**C**

**B**


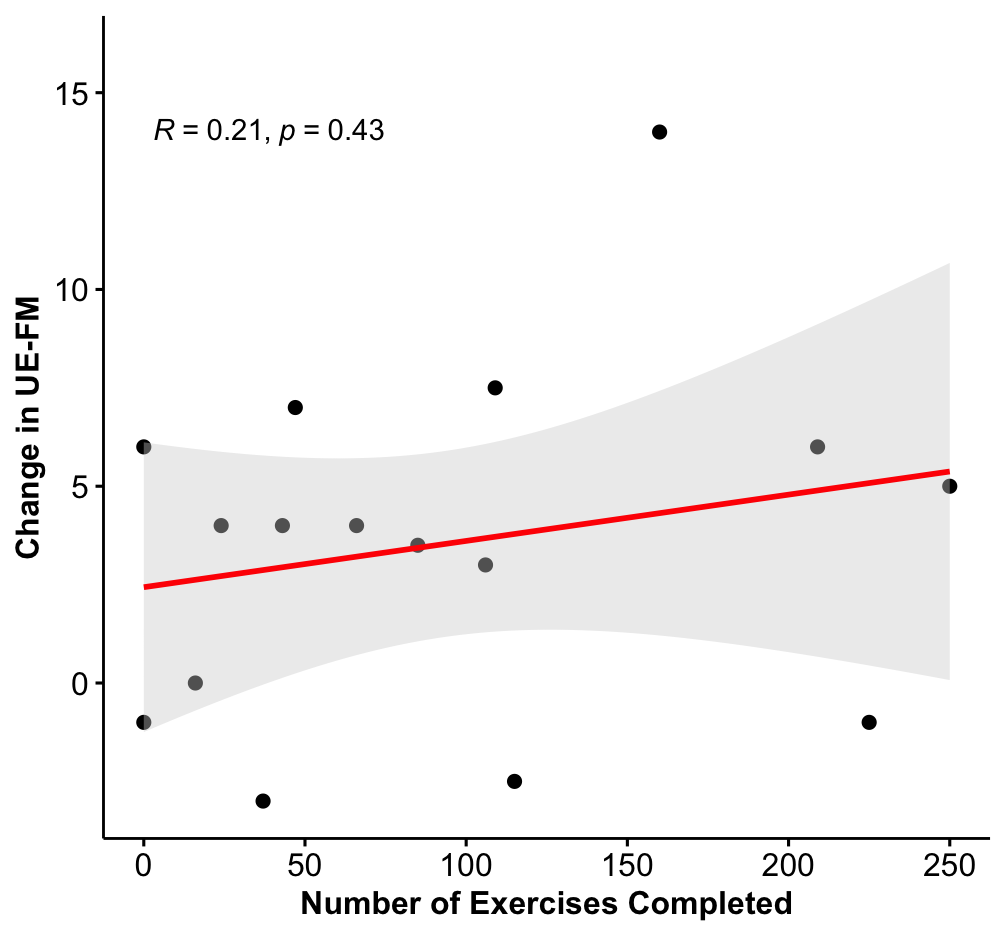

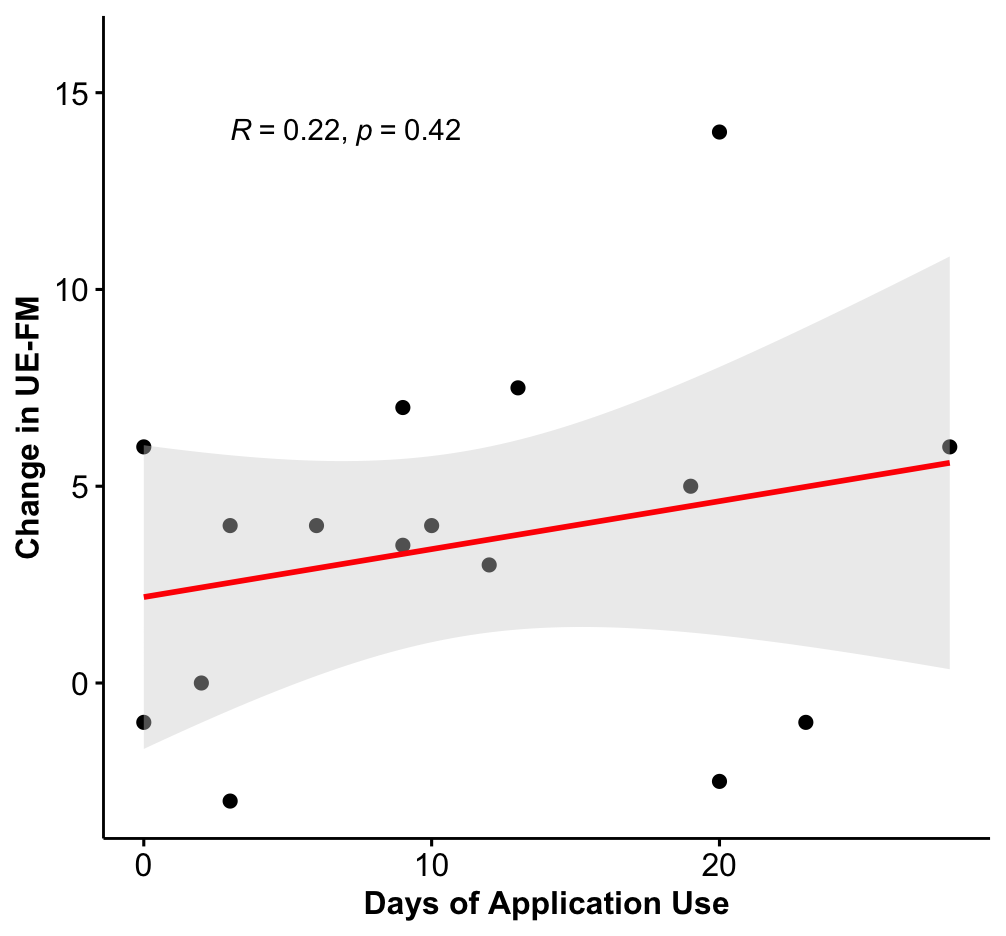

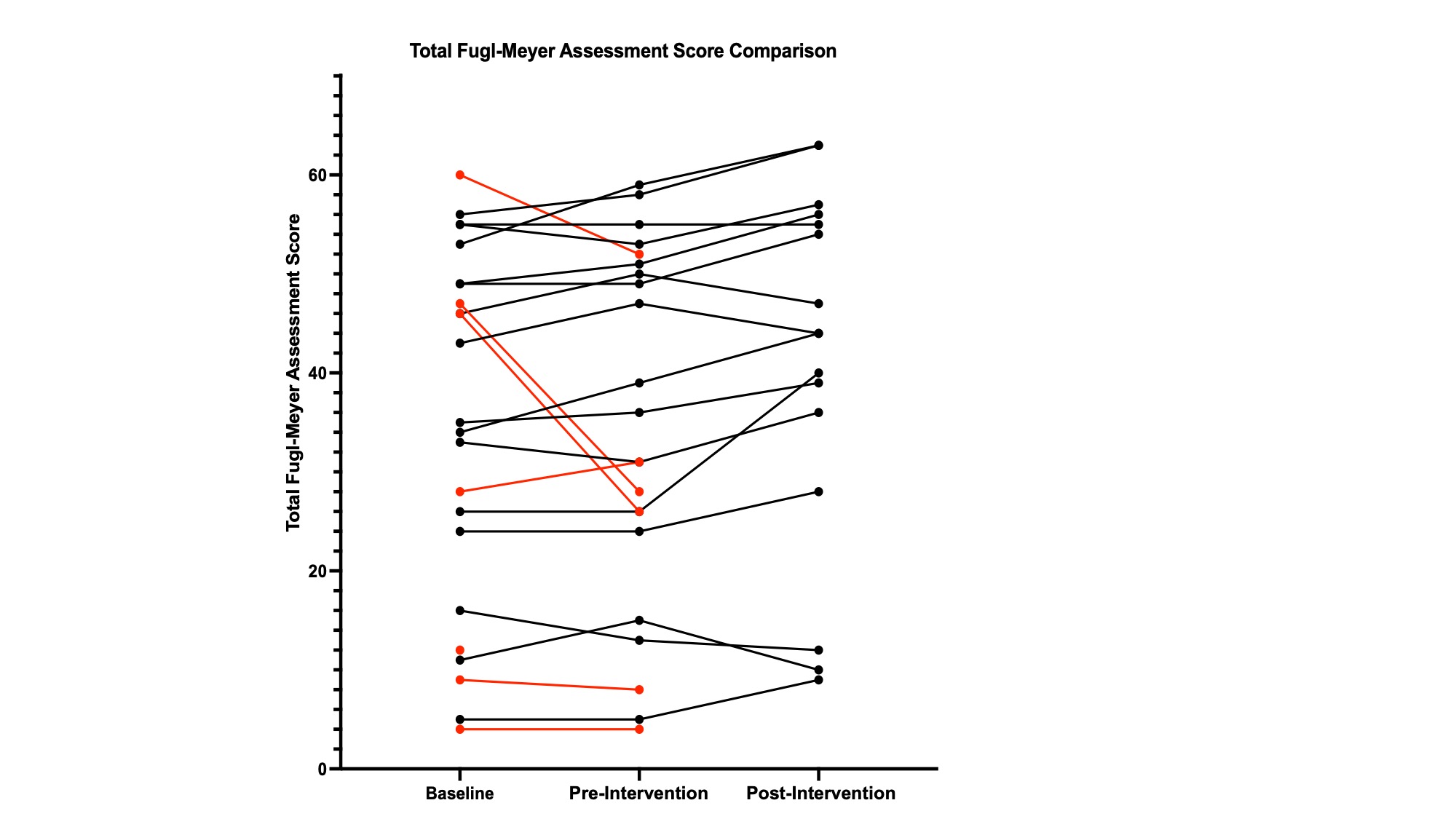


**A**
